# Supplementary material for: ATR, CHK1 and WEE1 inhibitors cause homologous recombination repair deficiency to induce synthetic lethality with PARP inhibitors
Source: Br J Cancer. 2024 Jul 4;131(5):905–17. doi: 10.1038/s41416-024-02745-0 (PMC11369084; doi:10.1038/s41416-024-02745-0)
Supplement: Supplementary file 1 — Figure S1 [file 41416_2024_2745_MOESM1_ESM.pdf]

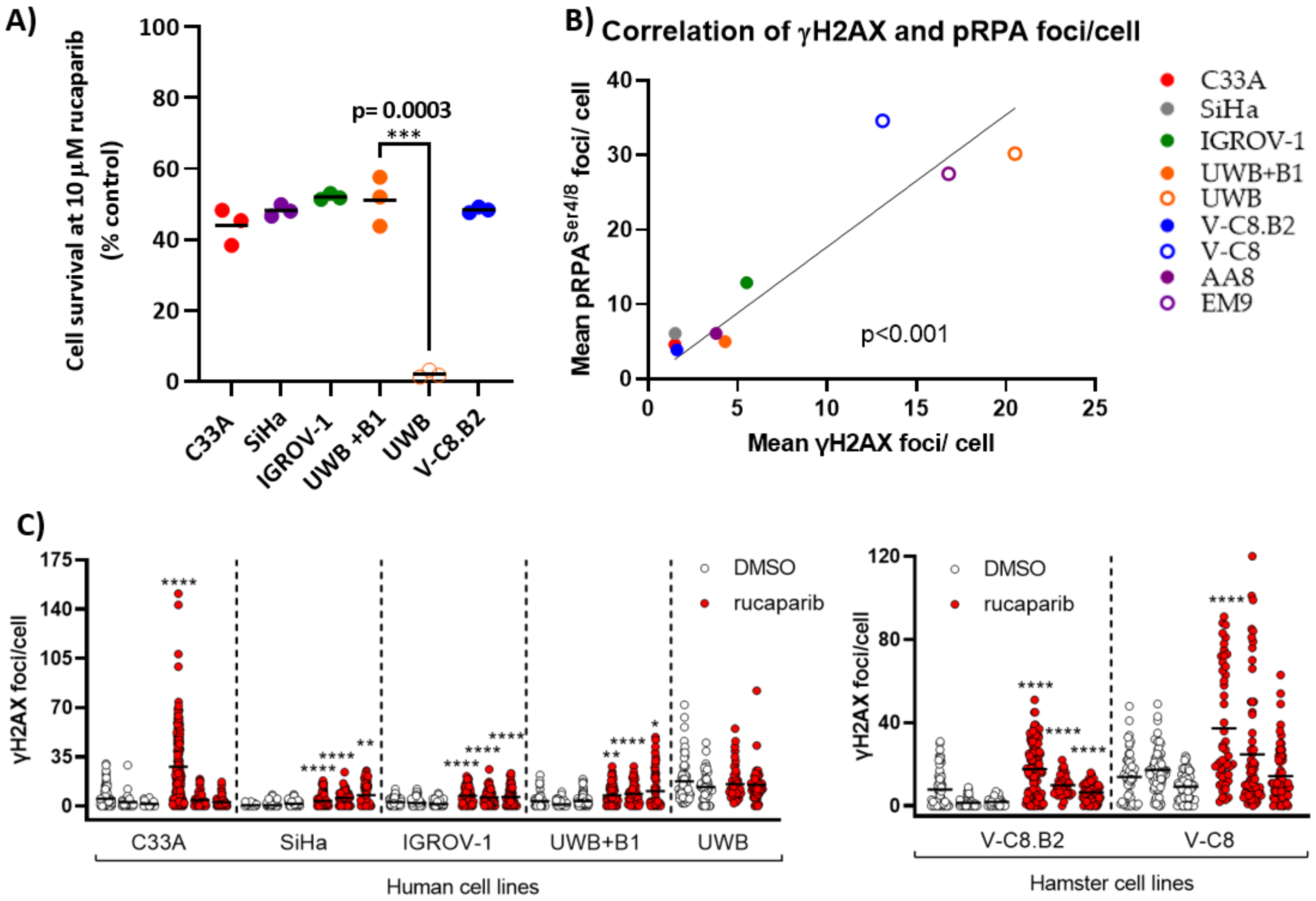

D)

Cell line	Mean LC50 ± S.D			
	Rucaparib [µM]	VE-821 [µM]	PF-477736 [nM]	MK-1775 [nM]
C33A	8.0 ± 1.2	1.7 ± 0.3	140.4 ± 23.0	254.7 ± 35.2
SiHa	8.2 ± 2.1	1.6 ± 0.6	125.5 ± 53.9	339.3 ± 40.2
IGROV-1	11.7 ± 0.3	1.8 ± 0.5	22.4 ± 0.9	121.4 ± 28.7
UWB+B1	12.3 ± 3.2	1.6 ± 0.6	43.7 ± 11.0	207.2 ± 8.0
UWB	0.19 ± 0.01	0.6 ± 0.09	27.3 ± 8.8	151.6 ± 33.9
V-C8.B2	8.8 ± 0.5	2.1 ± 0.2	87.5 ± 63.8	372.1 ± 150.2
V-C8	0.01 ± 0.003	1.9 ± 0.1	100.9 ± 36.3	454.5 ± 98.5

E)

	mean cell survival (% control) ± S.D						
	C33A	SiHa	IGROV-1	UWB+B1	UWB	V-C8.B2	V-C8
1 µM VE-821	55.3 ± 5.8	58.9 ± 3.5	64.4 ± 4.8	61.3 ± 13.0	32.2 ± 2.5	64.6 ± 2.3	60.8 ± 1.0
50 nM PF-477736	81.0 ± 5.6	57.2 ± 5.3	30.1 ± 2.1	44.6 ± 9.5	27.5 ± 2.9	61.5 ± 9.8	56.0 ± 7.2
100 nM MK-1775	84.6 ± 15.1	63.5 ± 2.3	51.4 ± 3.9	82.7 ± 11.6	74.9 ± 20.2	71.2 ± 11.2	88.6 ± 9.5

Supplementary figure 1. A. Colony formation-based cell survival following exposure of cells to 10 µM rucaparib for 24 h. Data, percent survival relative to vehicle (DMSO) control are 3 independent experiments. **B.** Correlation of mean pRPAS4/8 foci/cell with mean γH2AX foci/cell. Mean value was from 3 independent experiments and significance was calculated in Graph pad Prism 9.0. **C.** Scatter plots of γH2AX foci/cell values for 3 independent experiments for all cells except UWB, where each column represents an individual experiment. Collated data is shown in Figure 1C. Significance of increase from DMSO control cells to those treated with 10 µM rucaparib was calculated with Graph pad Prism 9.0 * $p<0.05$, ** $p<0.01$, *** $p<0.0001$. **D.** Mean LC50 data for rucaparib, VE-821, PF-477736 and MK-1775, calculated from data shown in Fig 1A and Fig 3A following exposure to compounds for 24 h. Data are mean ± SEM of 3 independent experiments. **E.** The mean survival of cells (% control) was calculated from single agent checkpoint kinase inhibitor cytotoxicity data, with the view of use in combination studies, whilst ensuring limited excessive toxicity. Data are mean ± SEM of 3 independent experiments.
