# Supplementary material for: ATR, CHK1 and WEE1 inhibitors cause homologous recombination repair deficiency to induce synthetic lethality with PARP inhibitors
Source: Br J Cancer. 2024 Jul 4;131(5):905–17. doi: 10.1038/s41416-024-02745-0 (PMC11369084; doi:10.1038/s41416-024-02745-0)
Supplement: Supplementary file 2 — Figure S2 [file 41416_2024_2745_MOESM2_ESM.pdf]

A)

		C33A	SiHa	IGROV-1	UWB+B1	V-C8.B2
**Rucaparib single agent**	Mean cell survival at 10 µM rucaparib (%) ± S.D	24.0 ± 5.1	45.9 ± 15.2	52.1 ± 0.8	48.3 ± 4.2	45.6 ± 3.9
**+ 1 µM VE-821**	Mean cell survival at 10 µM rucaparib (%) ± S.D	12.7 ± 6.8	17.1 ± 9.7	33.2 ± 4.4	26.6 ± 5.3	10.0 ± 3.7
	Mean potentiation factor ± S.D	2.5 ± 2.0	3.5 ± 2.9	1.6 ± 0.2	1.8 ± 0.2	5.1 ± 1.9
**+ 50 nM PF-477736**	Mean cell survival at 10 µM rucaparib (%) ± S.D	8.4 ± 6.9	20.9 ± 2.8	32.3 ± 1.8	42.7 ± 6.1	15.4 ± 3.6
	Mean potentiation factor ± S.D	3.9 ± 1.9	2.2 ± 0.6	1.6 ± 0.1	1.1 ± 0.2	3.0 ± 0.5
**+ 100 nM MK-1775**	Mean cell survival at 10 µM rucaparib (%) ± S.D	10.3 ± 5.6	24.9 ± 0.8	25.6 ± 2.7	25.1 ± 8.0	14.9 ± 3.5
	Mean potentiation factor ± S.D	2.6 ± 0.8	1.9 ± 0.7	2.1 ± 0.2	2.1 ± 0.7	3.2 ± 0.9

B)

AUC values of HRP cell lines

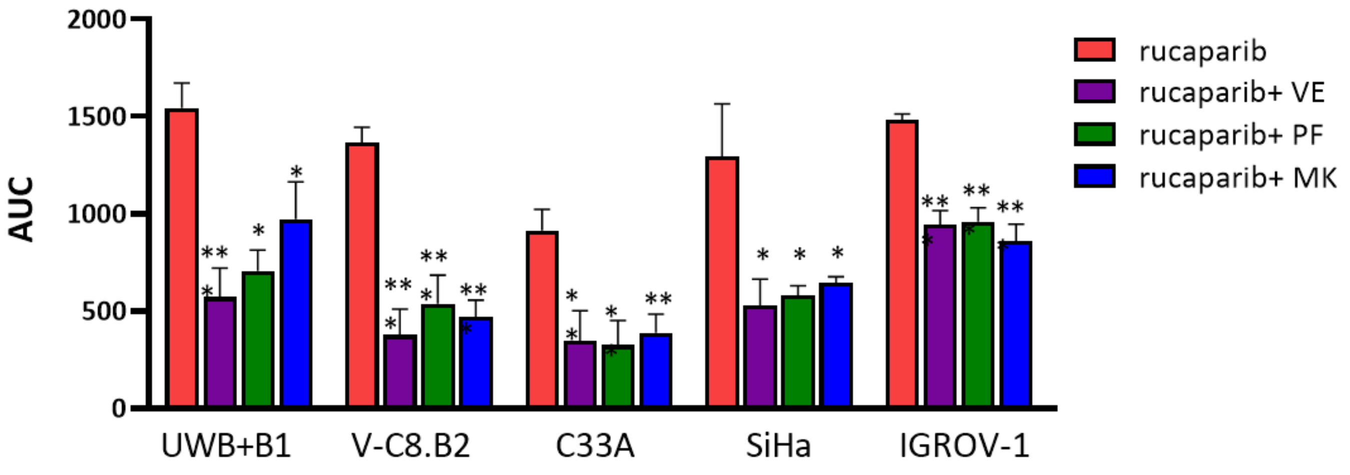

Supplementary figure 2. A. .Cells were exposed to rucaparib at indicated concentrations either as single agent or with the addition of 1 µM VE-821, 50 nM PF-477736 or 100 nM MK-1775 for 24 h prior to replacement with drug-free medium for 8-12 days to allow colony formation**.** Potentiation factor was calculated at by dividing mean % cell survival at 10 µM rucaparib single agent by mean % cell survival at 10 µM rucaparib with the addition of 1 µM VE-821, 50 nM PF-477736 or 100 nM Mk-1775. Data are mean ± SEM of 3 independent experiments. **B.** Area under curve (AUC) values were calculated with Graph pad Prism 9.0 software and data are the mean ± SEM of 3 independent experiments. Statistical analysis of the difference in AUC values between rucaparib single agent and with combination of VE-821, PF-477736 or MK-1775 was calculated with Graph pad Prism 9.0, * p<0.05, ** p<0.01, *** p<0.0001.
