# Supplementary material for: ATR, CHK1 and WEE1 inhibitors cause homologous recombination repair deficiency to induce synthetic lethality with PARP inhibitors
Source: Br J Cancer. 2024 Jul 4;131(5):905–17. doi: 10.1038/s41416-024-02745-0 (PMC11369084; doi:10.1038/s41416-024-02745-0)
Supplement: Supplementary file 3 — Figure S3 [file 41416_2024_2745_MOESM3_ESM.pdf]

A)

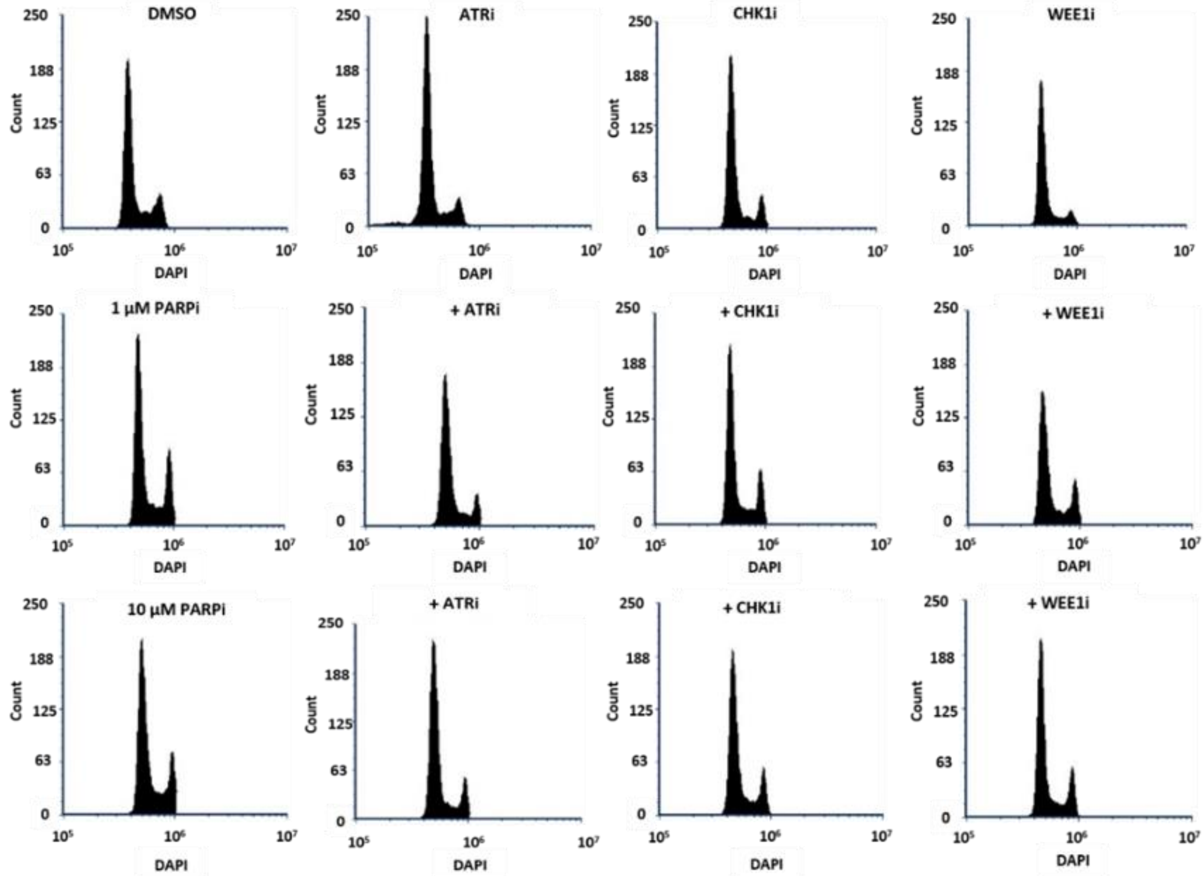

B)

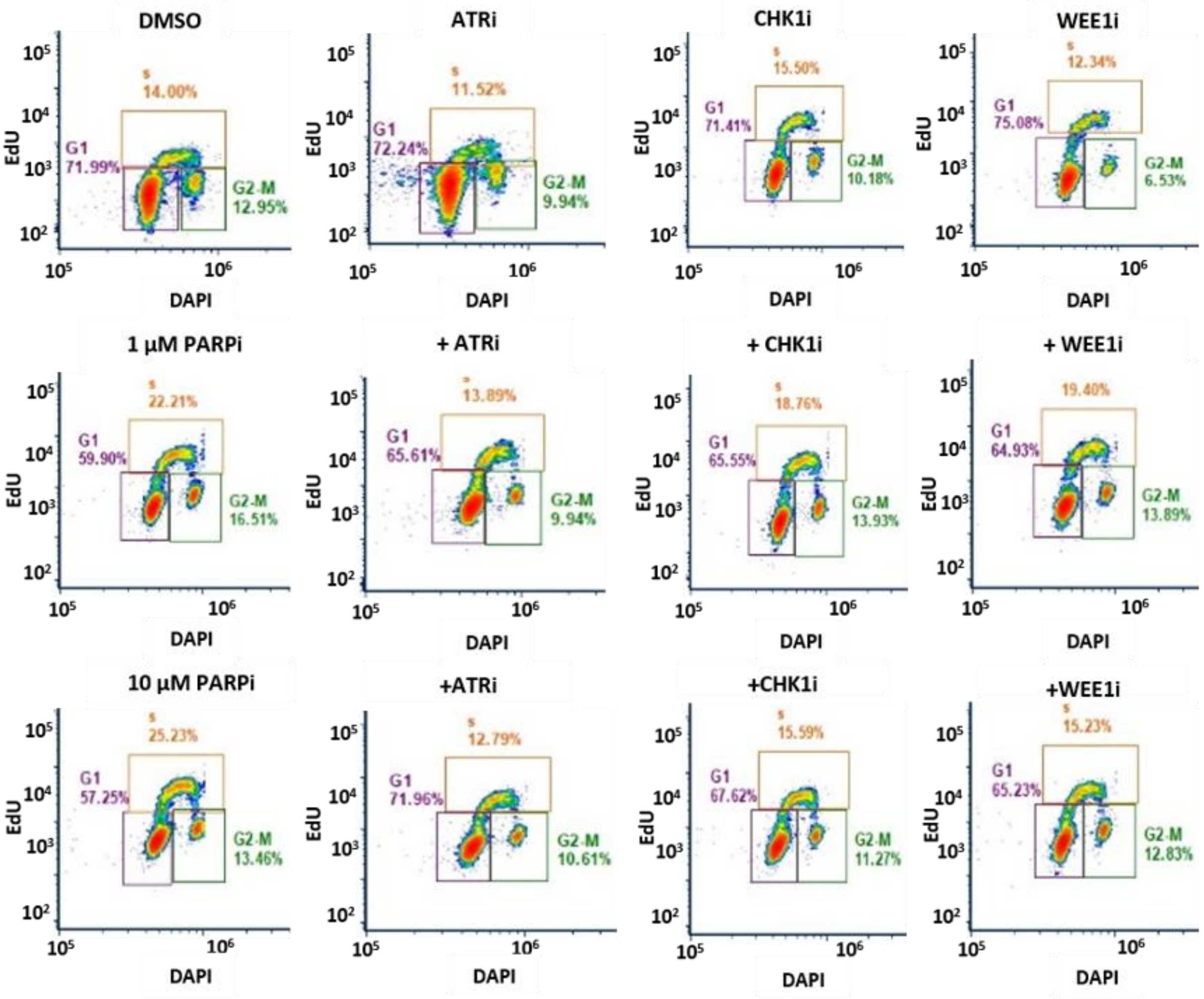

Supplementary figure 3. A. Histogram plots and **B.** density plots of cell cycle analysis of human ovarian cancer IGROV-1 and UWB paired cells following 24 h exposure to rucaparib single agent and in combination with VE-821 (1 µM), PF-477736 (50 nM) and MK-1775 (100 nM). Collated data of 3 independent experiments in IGROV-1 cells and mean of 2 independent experiments in UWB paired cells mean± SEM of is shown in figure 4A.
