# Supplementary material for: ATR, CHK1 and WEE1 inhibitors cause homologous recombination repair deficiency to induce synthetic lethality with PARP inhibitors
Source: Br J Cancer. 2024 Jul 4;131(5):905–17. doi: 10.1038/s41416-024-02745-0 (PMC11369084; doi:10.1038/s41416-024-02745-0)
Supplement: Supplementary file 4 — Figure S4 [file 41416_2024_2745_MOESM4_ESM.pdf]

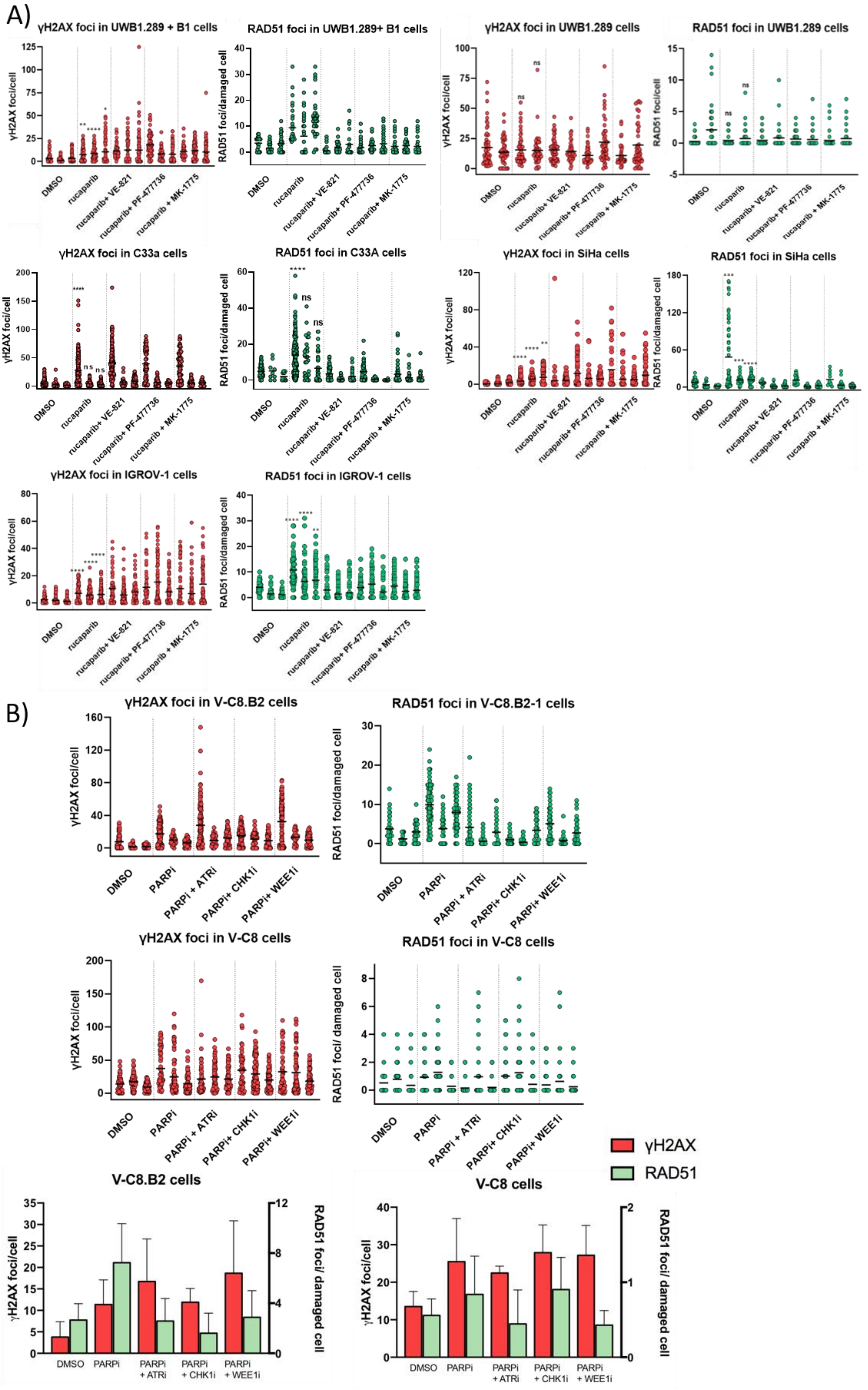

Supplementary figure 4. Scatter plots showing individual experiments pooled in figure 4B-D, with each column representing an independent experiment in **A.** UWB+B1, UWB, C33A, SiHa and IGROV-1 human cell lines and **B.** mouse fibroblast V-C8 paired cells. 3 independent experiments are shown in cells treated with 10 µM rucaparib single agent and with the addition of 1 µM VE-821, 50 nM PF-477736 or 100 nM MK-1775 for 24 h prior to fixation.
