# Supplementary material for: ATR, CHK1 and WEE1 inhibitors cause homologous recombination repair deficiency to induce synthetic lethality with PARP inhibitors
Source: Br J Cancer. 2024 Jul 4;131(5):905–17. doi: 10.1038/s41416-024-02745-0 (PMC11369084; doi:10.1038/s41416-024-02745-0)
Supplement: Supplementary file 5 — Figure S5 [file 41416_2024_2745_MOESM5_ESM.pdf]

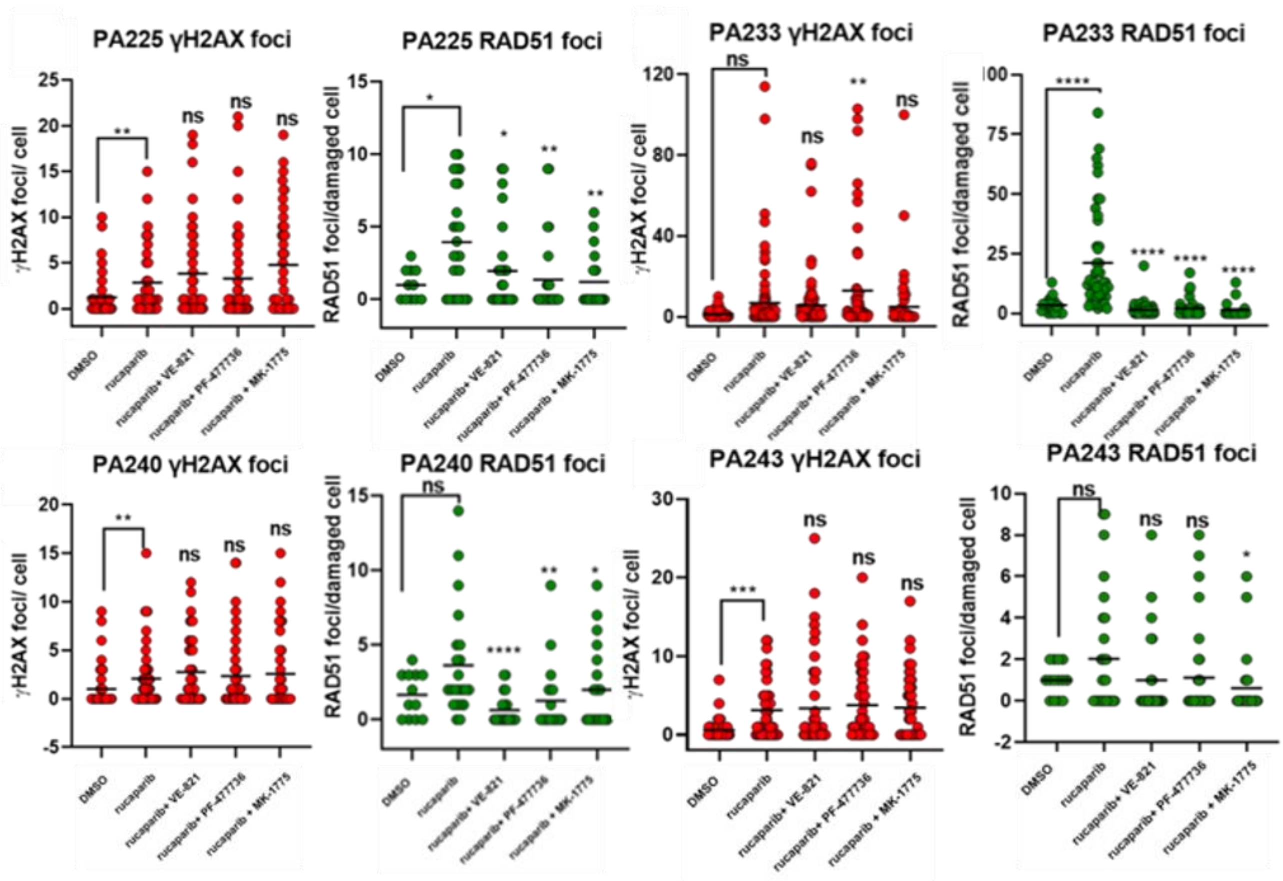

Supplementary figure 5. Scatter plots showing γH2AX and RAD51 foci/cell in patient-derived ascites samples. Data of mean foci/cell are shown in figure 5G. Data are one experiment per primary culture.
