# Supplementary material for: ATR, CHK1 and WEE1 inhibitors cause homologous recombination repair deficiency to induce synthetic lethality with PARP inhibitors
Source: Br J Cancer. 2024 Jul 4;131(5):905–17. doi: 10.1038/s41416-024-02745-0 (PMC11369084; doi:10.1038/s41416-024-02745-0)
Supplement: Supplementary file 6 — Figure S6 [file 41416_2024_2745_MOESM6_ESM.pdf]

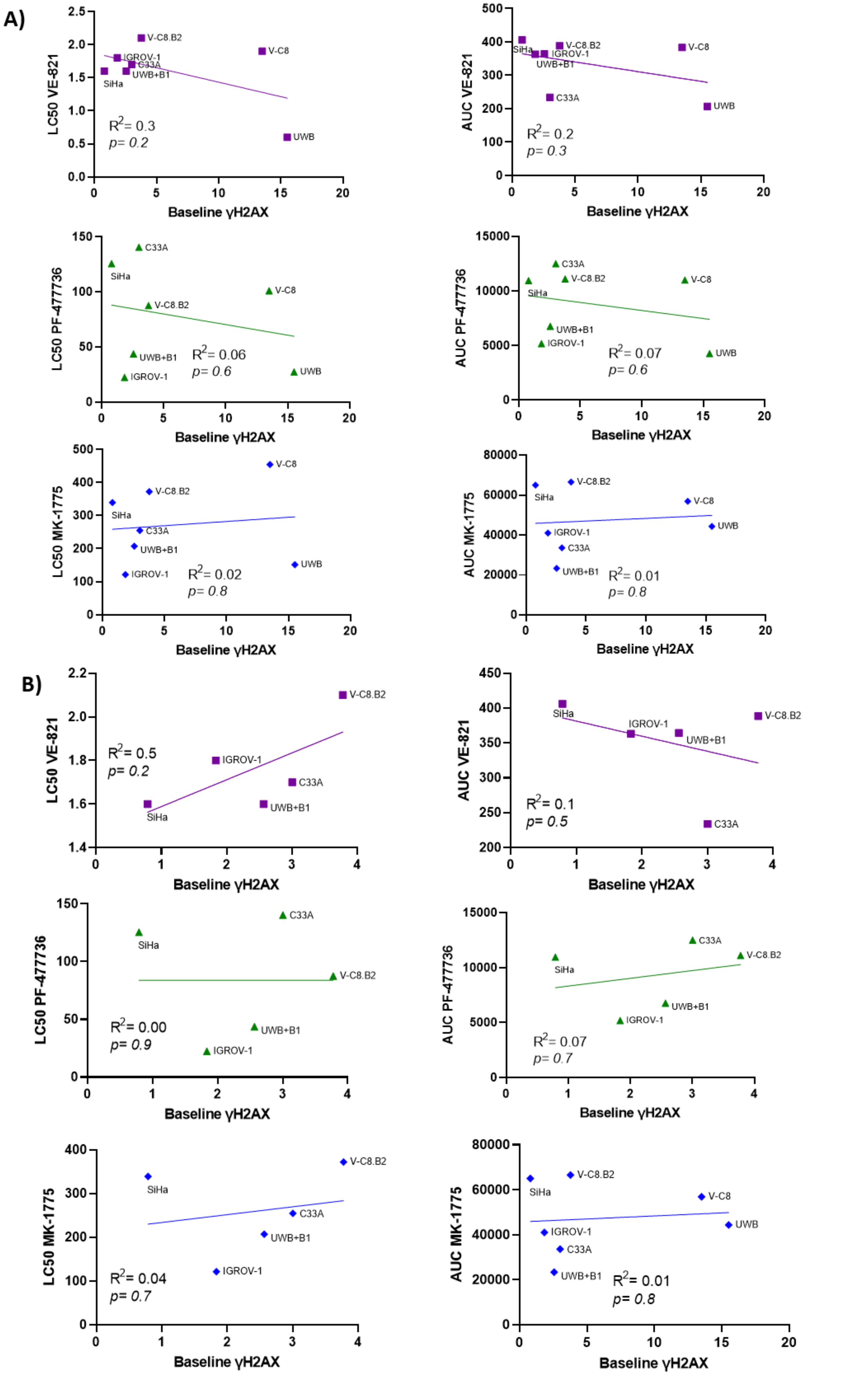

Supplementary figure 6. Assessing basal RS (γH2AX) as a determinant of sensitivity to single agent VE-821, PF-477736 and MK-1775 with **A.** all cell lines including HRD UWB and V-C8 cells and **B.** HRP cell lines, by correlating mean basal γH2AX with LC50 and AUC values of single agent checkpoint kinase inhibitors. Data are mean of 3 independent experiments.
