# Supplementary material for: ATR, CHK1 and WEE1 inhibitors cause homologous recombination repair deficiency to induce synthetic lethality with PARP inhibitors
Source: Br J Cancer. 2024 Jul 4;131(5):905–17. doi: 10.1038/s41416-024-02745-0 (PMC11369084; doi:10.1038/s41416-024-02745-0)
Supplement: Supplementary file 7 — Figure S7 [file 41416_2024_2745_MOESM7_ESM.pdf]

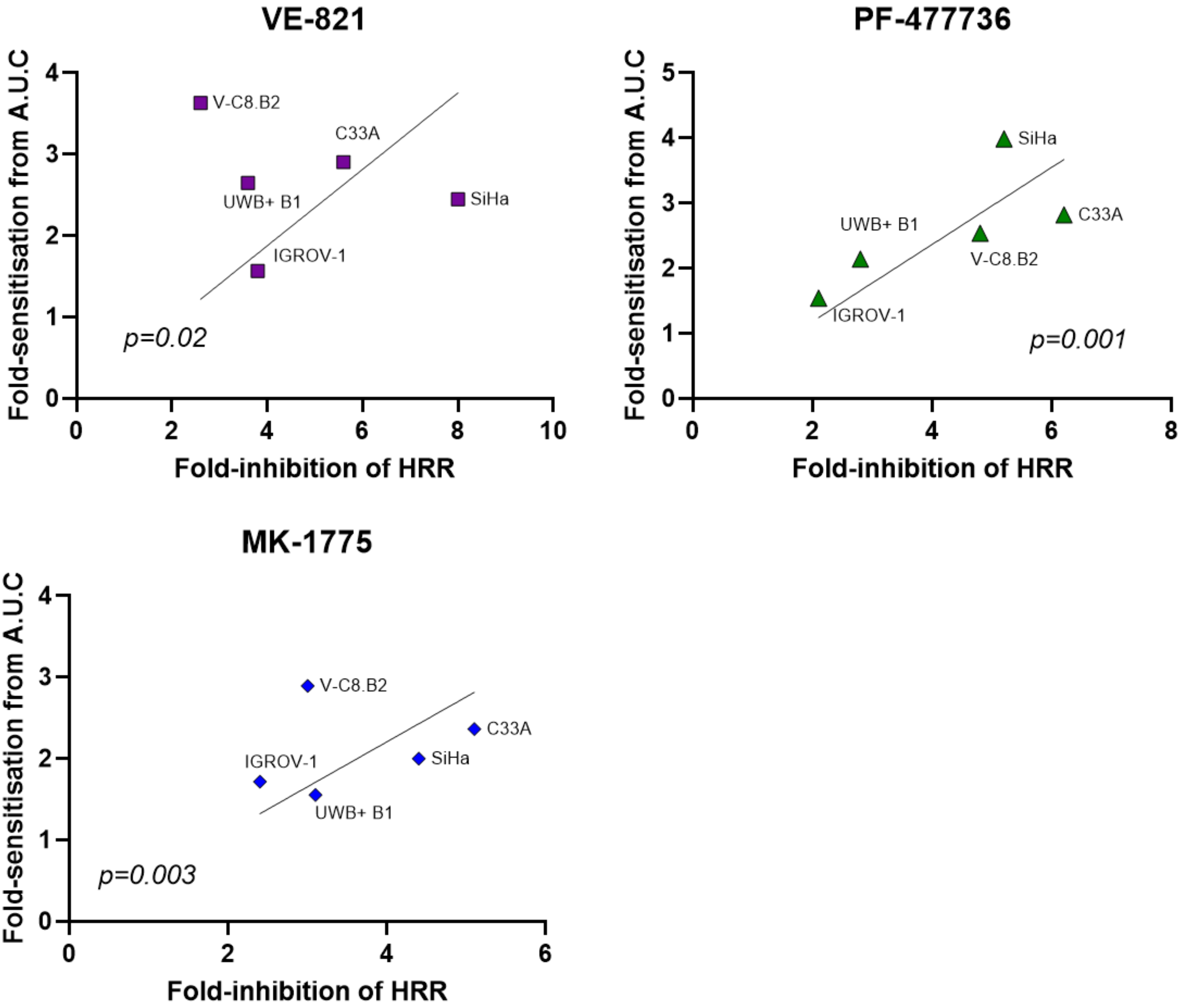

Supplementary figure 7. Fold-sensitisation of HRP cells by addition of 1 µM VE-821, 50 nM PF-477736 and 100 nM MK-1775 as determined from AUC values vs fold-inhibition of HRR caused by the addition of each of the checkpoint kinase inhibitors. Data plotted are mean of 3 independent experiments.
